# The Paradoxical Signals of Two TrkC Receptor Isoforms Supports a Rationale for Novel Therapeutic Strategies in ALS

**DOI:** 10.1371/journal.pone.0162307

**Published:** 2016-10-03

**Authors:** Fouad Brahimi, Mario Maira, Pablo F. Barcelona, Alba Galan, Tahar Aboulkassim, Katrina Teske, Mary-Louise Rogers, Lisa Bertram, Jing Wang, Masoud Yousefi, Robert Rush, Marc Fabian, Neil Cashman, H. Uri Saragovi

**Affiliations:** 1 Lady Davis Institute-Jewish General Hospital, Translational Center, McGill University, Montréal, QC, Canada; 2 Department of Pharmacology and Therapeutics, McGill University, Montréal, QC, Canada; 3 Department of Biochemistry. McGill University, Montréal, QC, Canada; 4 Flinders University, Department of Human Physiology, Centre for Neuroscience, Adelaide, Australia; 5 University of British Columbia. Brain Research Centre, Vancouver, Canada; Rutgers University, UNITED STATES

## Abstract

Full length TrkC (TrkC-FL) is a receptor tyrosine kinase whose mRNA can be spliced to a truncated TrkC.T1 isoform lacking the kinase domain. Neurotrophin-3 (NT-3) activates TrkC-FL to maintain motor neuron health and function and TrkC.T1 to produce neurotoxic TNF-α; hence resulting in opposing pathways. In mouse and human ALS spinal cord, the reduction of miR-128 that destabilizes TrkC.T1 mRNA results in up-regulated TrkC.T1 and TNF-α in astrocytes. We exploited conformational differences to develop an agonistic mAb 2B7 that selectively activates TrkC-FL, to circumvent TrkC.T1 activation. In mouse ALS, 2B7 activates spinal cord TrkC-FL signals, improves spinal cord motor neuron phenotype and function, and significantly prolongs life-span. Our results elucidate biological paradoxes of receptor isoforms and their role in disease progression, validate the concept of selectively targeting conformational epitopes in naturally occurring isoforms, and may guide the development of pro-neuroprotective (TrkC-FL) and anti-neurotoxic (TrkC.T1) therapeutic strategies.

## Introduction

Neurotrophins play a key role in the life, maintenance, phenotype, and function of adult neurons [1]. Specifically for motor neurons, Brain-derived neurotrophic factor (BDNF) and Neurotrophin-3 (NT-3) regulate survival, excitability, axon conduction velocity, and morphology [2] through activation of their respective receptor tyrosine kinases (RTKs) TrkB and TrkC [2,3].

Motor neurons degenerate in Amyotrophic lateral sclerosis (ALS), spinal muscular atrophy (SMA), and spinal cord injury (SCI) [4], and neurotrophic strategies that delay or prevent motor neuron death and rescue motor neuron function may be beneficial. Experimental ALS therapy required very high neurotrophin protein concentrations by intrathecal injections or expression by viral vectors [5] or a combination of at least two growth factors [6–8].

While the rationale of using BDNF and NT-3 as drugs for ALS seems strong, they have failed clinically [3]. There are several reasons postulated for the failure. One problem is that neurotrophins have a short half-life *in vivo* and may not reach the target tissue. A second problem relates to the vast expanse of tissue that needs to be reached, from peripheral nerve terminals to spinal cord, because TrkB and TrkC targets are distributed throughout the motor neuron. Activation of receptors at the neuromuscular junction in the periphery *versus* receptors at the neuronal cell body in the spinal cord result in transduction of different signals (neuritogenic *versus* neurotrophic pathways respectively) [9]. A neuroprotective therapy would require activation of both receptors pools.

A third problem is that NT-3 not only binds to pro-survival receptor full length TrkC (TrkC-FL), but also binds to receptors that have pro-inflammatory or neurodegenerative functions: p75^NTR^ and truncated isoforms of TrkC (TrkC.T1). The unintended p75^NTR^ target is expressed in most neurons, glia, and many other cell types. The p75^NTR^ functions to execute the axonal pruning and cell death required during embryonic development [10], and it is up-regulated in ALS [11]. When neurotrophins are used as drugs it is essential to circumvent activation of p75^NTR^, and this has been achieved *in vivo* using neurotrophin mutants or p75-blocking strategies [12–14]. The unintended TrkC.T1 truncated isoform target is an mRNA splice variant that lacks the kinase intracellular domain, but retains the ectodomain and transmembrane primary sequence identical to TrkC-FL [12]. While TrkC-FL has trophic activity, multiple lines of evidence (biochemical, cell biological, genetic, and pharmacological) indicate that TrkC.T1 activates Rac1 and can be deleterious to neurons [15,16]. However, discriminating between TrkC-FL and TrkC.T1 activity is difficult because NT-3 binds to both isoforms with equal affinities [17].

To circumvent the problems associated with the use of NT-3 as a therapeutic (binding p75^NTR^ and TrkC.T1, short half-life, not reaching all receptor pools), we used antibody-based ligands [3,17–19] to develop a selective agonist that can discriminate between TrkC-FL and TrkC.T1 isoforms even though these receptors have identical ectodomain primary sequences. Selectivity was achieved by targeting disulfide-stabilized ectodomain secondary structures in TrkC-FL, structures which are absent in TrkC.T1 due to the influence of an intracellular neoepitope of TrkC.T1.

We also demonstrate that TrkC.T1 is up-regulated in mouse and human ALS, due to decreased miR-128, a miR that destabilizes TrkC.T1 mRNA. TrkC.T1 protein and mRNA are present in activated spinal cord astrocytes, and we show *ex vivo* that in an NT-3–dependent manner TrkC.T1 mediates up-regulation of TNF-α. Therefore, using the TrkC-FL selective agonist, and validate ligand-dependent activation of this target for therapy in a mouse model ALS.

Together, these data elucidate biological paradoxes of growth factor receptors and their isoforms in disease progression, validate the concept of selective targeting of conformational epitopes present in naturally occurring receptor isoforms of identical primary sequence, and validate selective activation of TrkC-FL as a therapeutic target in model of ALS.

## Results

### MAb 2B7 binds selectively to full-length TrkC

**Fig 1:**
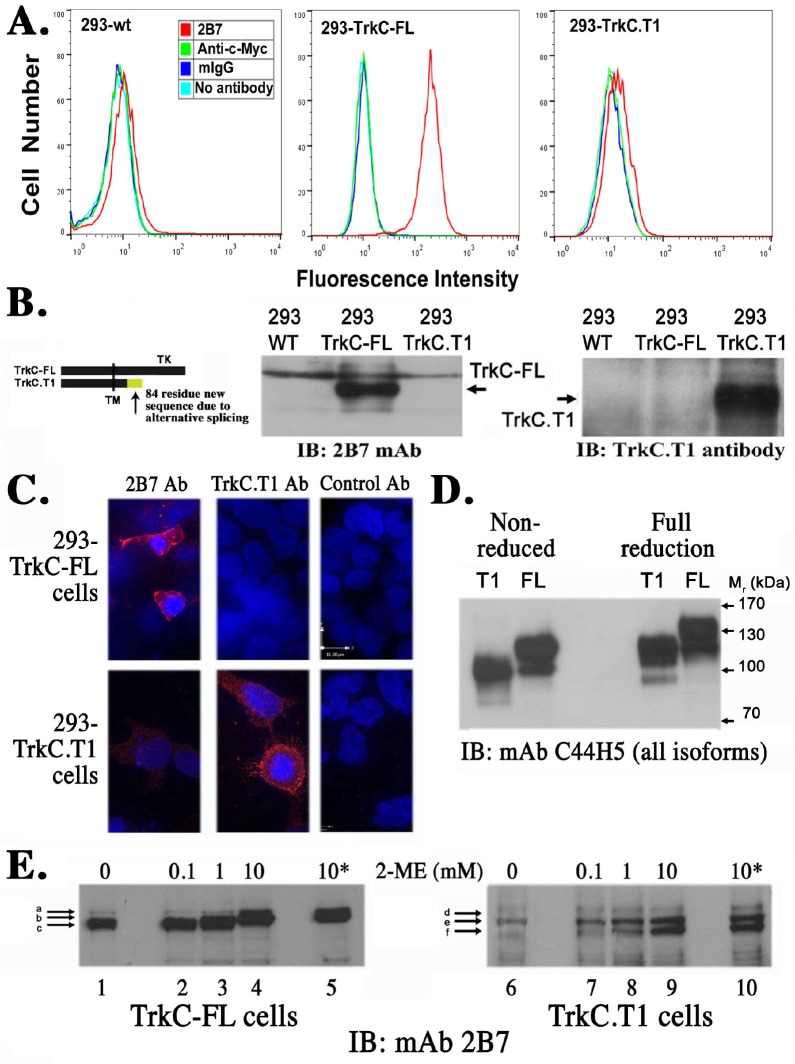
2B7 mAb binds only to full length TrkC. **(A)** HEK293-TrkC-FL or HEK293-TrkC.T1 cells were studied by FACScan. HEK293 wild type cells are negative control. MAb 2B7 (red histograms) binds to cell surface TrkC-FL protein, at mean channel fluorescence ~300. Several isotype-matched controls are shown for background, all at mean channel fluorescence ~10. No significant binding to the cell surface was detected using mAb 2B7 on NIH-TrkC.T1 cells, mean channel fluorescence ~15. **(B)** Non-reducing western blots of HEK293-TrkC-FL or HEK293-TrkC.T1 cells. MAb 2B7 only recognizes lysates form TrkC-FL cells. A control antibody 750 (against an intracellular neo-epitope that appears due to mRNA splicing) only recognizes TrkC.T1 and demonstrates that the cells express TrkC.T1 protein. **(C)** Fluorescent microscopy of 293-TrkC-FL or 293-TrkC.T1 cells immunostained with primary mAb 2B7, or antibody 750, or with non-binding mouse IgG control (red). DAPI counterstain for nuclei. Pictures were taken using a Leica confocal microscope with 63x magnification. MAb 2B7 only recognizes full length TrkC, whereas antibody 750 only recognizes TrkC.T1. (**D**) Lysates were prepared from HEK293-TrkC-FL or HEK293-TrkC.T1 cells and split as equal samples. For immunoblotting with mAb C44H5 (total TrkC). Samples were resolved in SDS-PAGE either non-reduced or fully reduced (50 mM 2-ME, with 5 min boiling). (**E**) Immunoblotting with mAb 2B7 (TrkC-FL specific). Samples were exposed to increasing amounts of reducing agent for 15’ at room temperature, and a replicate sample containing 10 mM 2-ME (10*) was also heated to 90°C for 1 min. Equal loading in all lanes was verified. Data representative of n = 3 independent experiments with equal results. The major specific bands are indicated by arrows and letters. The M_r_ of the bands on SDS-PAGE changes upon mild reduction.

MAb 2B7 and its Fab bind to an ectodomain epitope near the transmembrane domain [20]. FACScan studies demonstrated that mAb 2B7 binds to cells expressing surface TrkC-FL but does not bind (or binds very poorly) to cell surface TrkC.T1 (**Fig 1A**). Binding to the cell surface was measured on live HEK293 cells stably transfected to express equal numbers of either TrkC-FL or TrkC.T1 (cDNAs were expressed so there is no possible mRNA truncation). 2B7 binding to TrkC-FL and not to TrkC.T1 was evaluated after transfection of human and rat cDNAs, with similar results.

These FACScan studies are quantitative, as they use a homogeneous population expressing a single receptor isoform, they yield single bell-shaped histograms that can detect ~1,000 receptors/cell, with data from 5,000 to 10,000 cells acquired per experiment (n>10 independent experiments), and in each independent experiment the TrkC-FL signal shifted >25-fold above background and the TrkC.T1 signal shifted <2-fold above background).

FACScan studies using rat rMC-1 cells endogenously expressing TrkC.T1 mRNA and protein showed undetectable binding by mAb 2B7, and previous studies demonstrated 2B7 binding to TrkC-FL endogenously expressed in rodent primary neuronal cultures [3]. The 2B7 epitope is at the juxtamembrane in the ectodomain, and both TrkC-FL and TrkC.T1 have the primary sequence of the epitope (**Fig 1B**). Previously, we showed that in western blots mAb 2B7 bound to TrkC-FL under non-reducing conditions but not under fully reducing conditions [21,22]. Here, we show in western blot studies using non-reduced samples that mAb 2B7 binds to 293-TrkC-FL whole cell lysates but it does not bind to 293-TrkC.T1 whole cell lysates. In contrast, a control 750 antibody against the TrkC.T1 intracellular neo-epitope binds to 293-TrkC.T1 whole cell lysates but not 293-TrkC-FL whole cell lysates (**Fig 1B**). In immunofluorescence studies mAb 2B7 binds to 293-TrkC-FL and antibody 750 binds to 293-TrkC.T1 (**Fig 1C**) without significant cross-reactivity. These studies were replicated in cells expressing TrkC-FL or TrkC.T1 after transfection with human, mouse or rat cDNAs, with identical results (data not shown).

Together, these data indicate that mAb 2B7 binds specifically to the ectodomain of cell surface TrkC-FL from mouse, rat, and human species, but it does not bind to any TrkC.T1 species whether it is expressed after transfection or expressed endogenously.

### Receptor conformation determines ligand selectivity

MAb 2B7 binding is sensitive to full reduction of TrkC-FL (75 mM DTT, 5 min 100°C), suggesting that a disulfide bond-stabilized secondary structure is required for proper display of the epitope [23]. Both TrkC-FL and TrkC.T1 proteins have disulfide bonds. TrkC-FL migrates in SDS-PAGE as M_r_ ~15 kDa smaller than when fully reduced (M_r_ ~120 kDa and ~135 kDa bands respectively), and TrkC.T1 migrates in SDS-PAGE as M_r_ ~25 kDa smaller than when fully reduced (M_r_ ~100 kDa and ~125 kDa bands respectively), as detected using antibody C44H5 that binds to all TrkC isoforms (**Fig 1D**). TrkC.T1 migrates faster due to the truncation, and both TrkC-FL and TrkC.T1 migrate more slowly in their reduced state due to the “relaxed” state of the proteins. The minor bands detected by C44H5 likely correspond to differentially glycosylated receptors [23].

Both TrkC-FL and TrkC.T1 were subjected to mild to intermediate reducing conditions (0, 0.1, 1.0 and 10 mM DTT ± 1 min 90°C). These conditions preferentially reduce disulfide bonds exposed on the protein surface. MAb 2B7 detects non-reduced TrkC-FL as a single band of M_r_ ~120 kDa (**Fig 1E lane 1**), and mild reduction generates a “relaxed” protein M_r_ ~125 kDa, and intermediate reduction yields a M_r_ ~135 kDa band (**Fig 1E, lanes 2–5**). These mild/intermediate reducing conditions do not affect the mAb 2B7 epitope in TrkC-FL, and therefore could be used to study “gain of epitope” in TrkC.T1. MAb 2B7 does not detect non-reduced TrkC.T1 (**Fig 1E lane 6**). Mild and intermediate reduction affords progressively improved detection of TrkC.T1 by mAb 2B7 (**Fig 1E, lanes 7–10**).

Together, the data show that 2B7 selectively binds to TrkC-FL, at an epitope sensitive to disulfide bond arrangements which differ between TrkC-FL and TrkC.T1, and which can be resolved by mild reduction of exposed Cys-Cys bonds at the protein surface.

**Fig 2:**
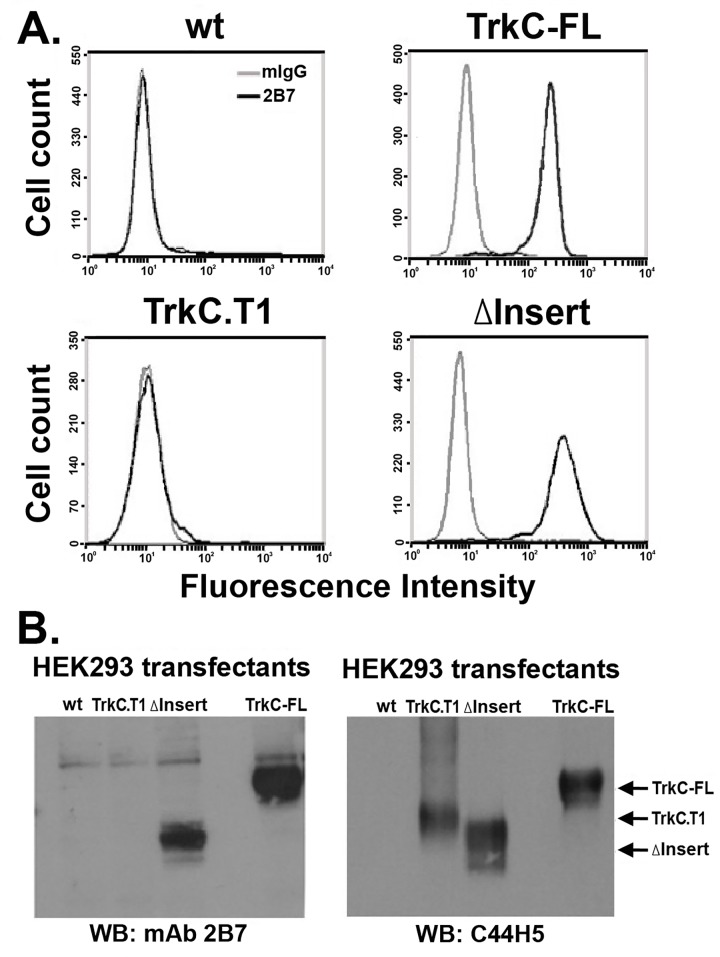
Deletion of the intracellular neo-epitope of TrkC.T1 affords 2B7 mAb recognition of the ectodomain. (**A**) The neo-epitope of TrkC.T1 (see Fig 1B) was deleted to generate TrkC.T1 ^Δinsert^. TrkC-FL, TrkC.T1, and TrkC.T1 ^Δinsert^ were each transfected in HEK293 cells. After drug selection stable transfectants or control cells were studied by FACScan binding assays using mAb 2B7. (**B**) Lysates of the same cells were studied by western blotting with mAb 2B7 or with mAb C44H5 (total TrkC).

A differential folding observed for TrkC-FL and TrkC.T1 (as defined by mAb 2B7 binding) suggest that either the loss of the intracellular TrkC-FL kinase domain or the gain of the 84 amino acid neo-epitope in the TrkC.T1 cytoplasmic tail affects how each protein is processed. These possibilities were evaluated (**Fig 2**) by expression of a TrkC.T1 cDNA construct where the 84 amino acid neo-epitope was deleted (TrkC.T1^Δinsert^). Expression of TrkC.T1 ^Δinsert^ resulted in proteins recognized by mAb 2B7 at the cell surface in FACScan assays (**Fig 2A**) and in non-reducing western blots (**Fig 2B**).

Together, these data demonstrate that a naturally occurring truncated isoform TrkC.T1 containing an intracellular neo-epitope has a new and different folding/conformation than TrkC-FL, not recognized by mAb 2B7. Deletion of the intracellular neo-epitope generates a structure that is recognized by mAb 2B7, like the TrkC-FL folding/conformation.

The three species we studied (human, rat and mouse) preserve the mRNA splicing that generates TrkC.T1, the neo-epitope of the intracellular domain of TrkC.T1, and the specific conformation of the ectodomain of each isoform (as defined by mAb 2B7). These data suggest an important function for TrkC.T1. The TrkC.T1 isoform transduces signals [23] linked to neurodegeneration [15], but its mechanism of action remained unclear. Given that NT-3 binds equally to TrkC-FL and to TrkC.T1, while mAb 2B7 is a selective ligand of TrkC-FL, we carried out functional studies to test the ligand-dependent mechanisms of action.

### TrkC.T1 induces TNF-α mRNA and protein in a ligand-dependent manner

**Fig 3:**
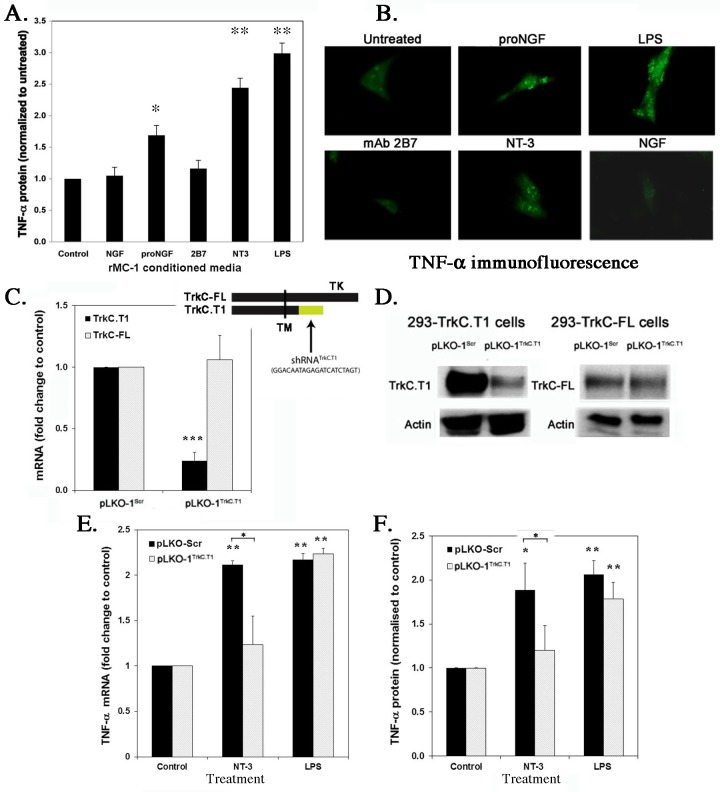
NT-3–dependent induction of TNF-α is TrkC.T1–mediated. (**A**) The glial rMC-1 cell line expressing endogenous TrkC-FL and TrkC.T1, and p75^NTR^ were treated for 6 hours (NGF 2 nM, proNGF 0.5 nM, 2B7 100 nM, NT-3 10 nM, LPS 1 μg/ml). From conditioned media the levels of soluble TNF-α protein were quantified by ELISA. Data are standardized to untreated control. Average ± SEM, n = 3 independent experiments each in triplicate. * p<0.05, ** p< 0.01 versus control. (**B**) The levels of cell-associated TNF-α protein were studied by immunofluorescence. (**C**) The mRNA levels of TrkC-FL and TrkC.T1 were quantified by PCR, after infection with lentivirus pLKO.Scr (scrambled control) or with pLKO.1 (expressing a unique shRNA targeting TrkC.T1 mRNA specifically). Results are normalized to reference GAPDH mRNA. Data are expressed as the mean + SEM; n = 3 independent experiments each in triplicate. The inset shows a western blot for TrkC-FL or TrkC.T1, from detergent lysates prepared from the cultures. (**D and E**) The levels of TNF-α mRNA or protein were quantified by PCR or by ELISA after infection with pLKO.Scr or pLKO.1. Data are standardized to untreated control. Average ± SEM, n = 3 independent experiments each in triplicate. (**F)** The levels of TNF-α mRNA in were assessed by quantitative PCR from spinal cord of ALS G93A *versus* wild type mice. Results were normalized to GAPDH. Data are expressed as the mean + SEM n = 8 independent spinal cords per group. **(A–F)** t-test was applied for statistical analysis. * p<0.05, ** p< 0.01 versus control. Brackets indicate differences between the indicated groups.

A rat glial cell line (rMC-1) endogenously expressing TrkC.T1 was studied after treatment with TrkC agonists. NT-3 significantly increased the secreted form of TNF-α (**Fig 3A**, ELISA of conditioned media, p<0.004 *versus* untreated cells), and increased the cell-bound form of TNF-α (**Fig 3B**, cell immunofluorescence). NT-3 up-regulated TNF-α to levels similar to positive control LPS (a ligand of Toll-like receptors), and to levels higher than positive proNGF, a control p75^NTR^ agonist known to increase TNF-α [23]. Several doses of mAb 2B7 did not increase TNF-α in conditioned media (**Fig 3A**) or in the cellular biomass (**Fig 3B**). The failure of mAb 2B7 to induce TNF-α via TrkC.T1 is consistent with mAb 2B7 not binding to TrkC.T1. Note that a full range of concentrations were tested, but to simplify the graphics only the optimal or biologically active concentrations of ligands (e.g. 100 nM mAb 2B7 verified to act as agonist of TrkC-FL) are shown.

NT-3 induction of TNF-α is dependent on TrkC.T1 expression, as shown by lack of TNF-α stimulation by NT-3 when TrkC.T1 expression is specifically silenced. TrkC.T1 mRNA was silenced in rMC-1 cells by infection with lentivirus PLKO.1^TrkC.T1^ expressing a unique shRNA sequence that destabilizes TrkC.T1 mRNA. Infection with PLKO.1^TrkC.T1^ reduced TrkC.T1 mRNA (**Fig 3C**) and protein (**Fig 3D**) without affecting TrkC-FL mRNA or protein, and control virus PLKO.1^Scrambled^ had no effect on expression of either TrkC.T1 or TrkC-FL mRNA and protein. Silencing TrkC.T1 mRNA and protein significantly prevented the NT-3–induction of TNF-α mRNA (**Fig 3E**) and protein (**Fig 3F**) (p<0.04 *versus* PLKO.1^Scrambled^). As internal control, in PLKO.1^TrkC.T1^ silenced cells the induction of TNF-α by LPS was not affected, and PLKO.1^Scrambled^ infection did not prevent induction of TNF-α by NT-3 or LPS treatment.

Together, the data indicate that TrkC.T1 regulates production of TNF-α in an agonist-dependent manner, and this could be a confounding factor when using NT-3 for ALS therapy. Therefore we studied the expression and the role of each TrkC isoform in ALS.

### TrkC.T1 up-regulation in spinal cord astrocytes in a mouse ALS model and human sporadic ALS is due to miR128 downregulation

**Fig 4:**
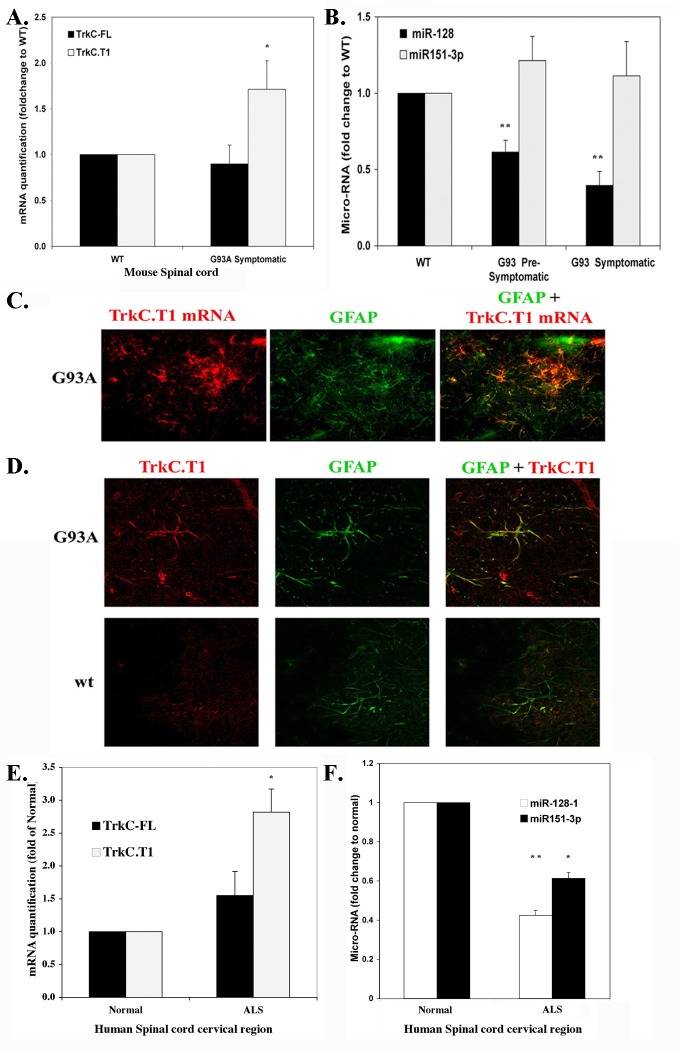
TrkC.T1 expression is up-regulated whereas miR-128 expression is down-regulated in the spinal cord of ALS G93A mice and in human SALS. **(A)**The mRNA levels of TrkC-FL and TrkC.T1 were assessed by quantitative PCR with SYBR Green (Quanta), normalized to GAPDH or β-tubulin III, for WT mice or ALS mice. Data are expressed as the mean + SEM (n = 8 spinal cord samples each group**(B)** The levels of miR-128 (regulator of TrkC.T1) and miR-151-3p (regulator of TrkC-FL) were measured by SYBR Green qRT-PCR (Quanta) and results were normalized to the reference RNU6 (small nuclear RNA). Data are expressed as the mean + SEM. **(C)** Combined fluorescent in situ mRNA hybridization (red, for TrkC.T1 mRNA) with immunofluorecence (green, GFAP marker). 40x magnification of 20 μm-thick sections of the lumbar spinal cord of G93A ALS mice. The probe specific for TrkC.T1 encompasses exons 13b & 14b as well as some of the 3'UTR. **(D)** Co-localization of TrkC.T1 protein (using anti-TrkC.T1 antibody 750) and GFAP in 12 μm thick sections from G93A ALS mice (top panels) or wild type mice (bottom panels). Pictures were taken using a Leica confocal microscope with 63x magnification. **(E)** The mRNA levels of TrkC-FL and TrkC.T1 were assessed by quantitative PCR with SYBR Green (Quanta), normalized to β-tubulin III, for human control (n = 3) or SALS (n = 4). Data are expressed as the mean + SEM. **(F)** From the same human samples the levels of miR-128 (regulator of TrkC.T1) and miR-151-3p (regulator of TrkC-FL) were measured by SYBR Green qRT-PCR (Quanta) and results were normalized to the reference RNU6 (small nuclear RNA). Data are expressed as the mean + SEM. **(A–F)** t-test was applied for statistical analysis. * p<0.05, ** p< 0.01 versus control.

In the spinal cord of symptomatic G93A ALS mice TrkC.T1 mRNA expression is significantly elevated, compared to wild type mice (p< 0.04). In contrast, expression of full-length TrkC (TrkC-FL) mRNA does not differ between ALS mice and wild type mice (**Fig 4A**). Standardization *versus* β-tubulin (a neuronal marker, to account for neuronal loss), or *versus* GAPDH (a ubiquitous marker) yields comparable results.

TrkC-FL and TrkC.T1 have non-overlapping 3'UTRs, and they are differentially regulated at the post-transcriptional level. The spliced TrkC.T1 mRNA is destabilized by micro-RNA miR128 [24]. In healthy wild type spinal cord miR128 is expressed. Pre-symptomatic ALS spinal cords (~100 days of age) have lower miR128, and symptomatic ALS spinal cords (~140 days of age) have even lower miR128 levels (p<0.003). In contrast, miR-151-3p that regulates full length TrkC-FL mRNA remains unchanged in wild type *versus* ALS mice (**Fig 4B**) and this miRNA serves as internal control. A miR128-promoted degradation would explain why healthy spinal cords have low or undetectable TrkC.T1 mRNA and protein, whereas reduced miR128 levels would lead to increased TrkC.T1 mRNA in the mouse model of ALS.

Similar results were obtained in spinal cords obtained post-mortem from human sporadic ALS (SALS, n = 4) *versus* non-ALS control (n = 3). In SALS there is a significant 2.84 ± 0.35 fold-increase in TrkC.T1 mRNA and a small non-significant elevation of TrkC-FL mRNA (standardized *versus* β-tubulin) (**Fig 4C**). Elevated TrkC.T1 mRNA in human SALS is associated with significantly reduced levels of miR128 (a known disruptor of TrkC.T1 mRNA) *versus* non-ALS control (**Fig 4D**) normalized to the reference RNU6 small nuclear RNA. In human SALS there was also a reduction in miR-151-3p (a known disruptor of TrkC-FL) *versus* non-ALS control and this resulted in a small and non-significant elevation of TrkC-FL mRNA.

The cells that express TrkC.T1 protein and mRNA in spinal cord were identified. Immunohistochemical studies using a specific TrkC.T1 antibody 750, showed expression in GFAP^+^ cells (astrocytes) of symptomatic ALS mice (**Fig 4E**), but not in Iba-1^+^ cells (microglia) or NeuN^+^ cells (neurons) (data not shown). In control wild type healthy spinal cords TrkC.T1 protein is virtually undetectable (**Fig 4E**), consistent with the very low number of GFAP^+^ activated astrocytes. These data were confirmed by *in situ* mRNA hybridization combined with immunohistochemical studies in spinal cord of symptomatic ALS mice (**Fig 4F**). The GFAP^+^ phenotype and the morphology of TrkC.T1 mRNA-expressing cells indicate that they are activated astrocytes.

Together, the data demonstrate that elevated TrkC.T1 mRNA and reduced miR128 are detected in the mutant SOD1 mouse model, and also in humans with ALS unrelated to SOD1 mutations (which represent the majority of clinical cases). In ALS, up-regulation of TrkC.T1 mRNA and protein occurs in astrocytes rather than neurons. The neurotoxic nature of elevated TNF-α in ALS has been documented, and our data provide a novel degenerative mechanism where NT-3 acting through TrkC.T1 can drive production of TNF-α and cause neurotoxicity.

### TrkC-FL activation by mAb 2B7 and Fabs is long-lived

**Fig 5:**
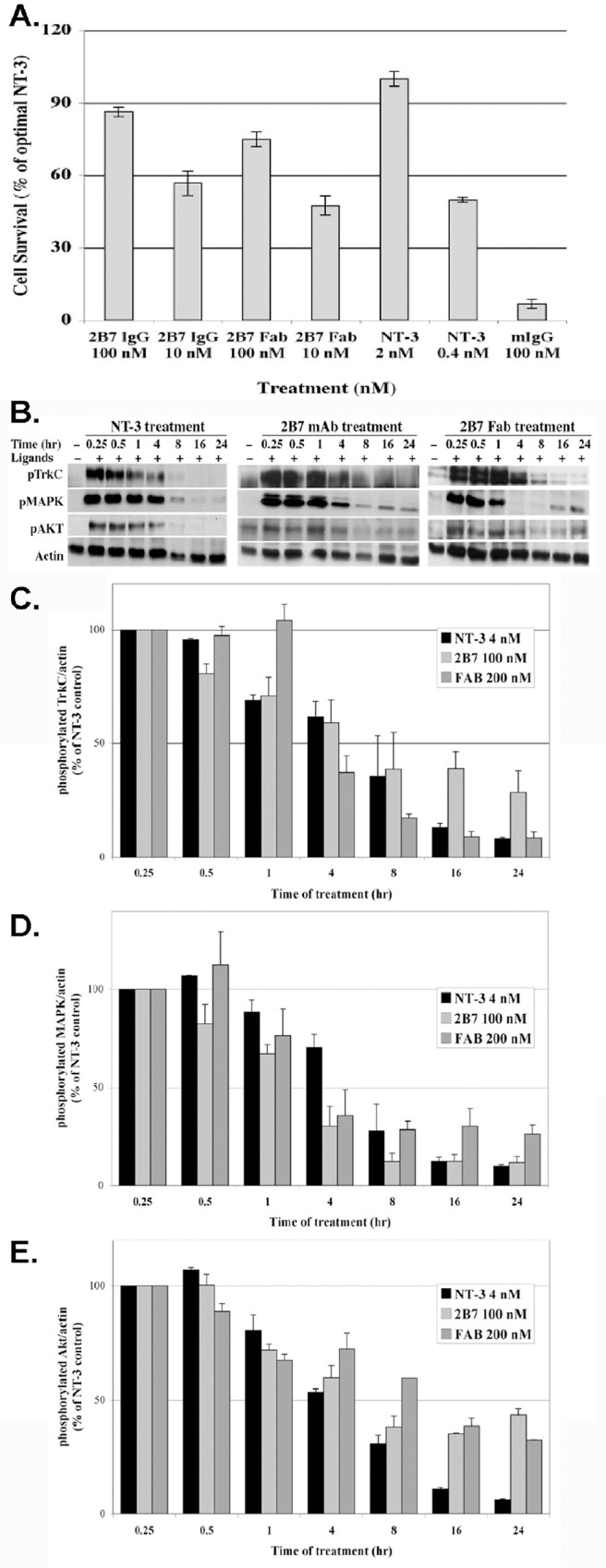
2B7 mAb and 2B7 Fab activate pro-survival activity and signaling pathways with sustained kinetics. **(A)** 2B7 and 2B7 Fabs protects NIH-TrkC cells from apoptotic death triggered by culture in serum-free conditions for 48 hours. NT-3 (2 nM) is the positive control, and was standardized as 100% since it is the optimal concentration. In cells that do not express TrkC, none of the ligands afford survival, indicating a TrkC-dependent signal (data not shown). Assay shown is representative of >5 independent experiments. Each assay n = 4 ± SEM.**(B)** 293-TrkC cells were cultured with 2B7 mAb (50 nM), 2B7 Fab (100 nM), NT-3 (4 nM) or control mIgG (100 nM) for the indicated times. Detergent lysates were analyzed by Western blotting for p-TrkC, p-MAPK, or p-Akt; standardized to actin. For these kinetic studies the molar concentrations of ligands result in comparable activation. Some of the exposures are not linear, this was intentional to show the longer time kinetics of some ligands. Quantifications were done from properly exposed gels. (**C**) p-TrkC densitomeric quantification + SEM of 3 independent experiments. (**D)** p-MAPK densitomeric quantification + SEM of 3 independent experiments. (**E**) p-AKT densitomeric quantification + SEM of 3 independent experiments. For visual clarity, the statistical differences are discussed in the text.

Previous work using neuronal cell lines and primary neuronal cultures showed that mAb 2B7 and 2B7 Fabs activate endogenously expressed TrkC-FL [17]. Here we show a detailed study of the kinetics of activation by these agonists. Biological (cell survival assays, **Fig 5A**) and biochemical (signal transduction assays, **Fig 5B**) show comparable activation by control NT-3, mAb 2B7, and 2B7 Fabs, which is sustained for up to 4 hr. In **Fig 5B** some of the exposures are not linear, this was intentional to show the longer time kinetics of some ligands. Quantifications were done from properly exposed gels and show that mAb 2B7 or 2B7 Fabs sustain the p-AKT signal significantly longer (p< 0.0002) than NT-3, with the signal persisting >24 hours (quantification in **Fig 5C, 5D and 5E**). In cellular controls, the mAb 2B7 and its Fabs do not activate signals in cells transfected to express a receptor family member TrkA or TrkB (data not shown).

Sustained activity is a known characteristic of the Trk receptor family and it is required for neuronal survival [25,26].

### 2B7 and 2B7 Fabs are stable *in vivo*

The poor specificity of NT-3 (binding to multiple receptors TrkC-FL, p75^NTR^, TrkC.T1), poor pharmacokinetics (circulation half-life under 2 minutes), and the inability to reach the different pools of receptors make NT-3 a poor therapeutic agent for ALS. On the other hand, mAb 2B7 has high TrkC-FL selectivity, does not bind to TrkC.T1 or p75^NTR^, and it does not induce increased TNF-α. Moreover, mAbs are known to be relatively stable in circulation.

For a pharmacokinetic study, 2B7 or 2B7 Fabs were administered intraperitoneally, and serum was prepared from blood samples collected over time. The presence of 2B7 or 2B7 Fabs in serum was quantified by ELISA against the original peptide epitope, as described [27,28]. Extrapolation from binding curves indicate that the half-lives of circulating and bioavailable 2B7 Fab and 2B7 mAb that are able to bind to the target were respectively ~36 hours and ~80 hours.

### 2B7 is present in spinal cord neurons and activates pro-survival signals in spinal cord *in vivo*

**Fig 6:**
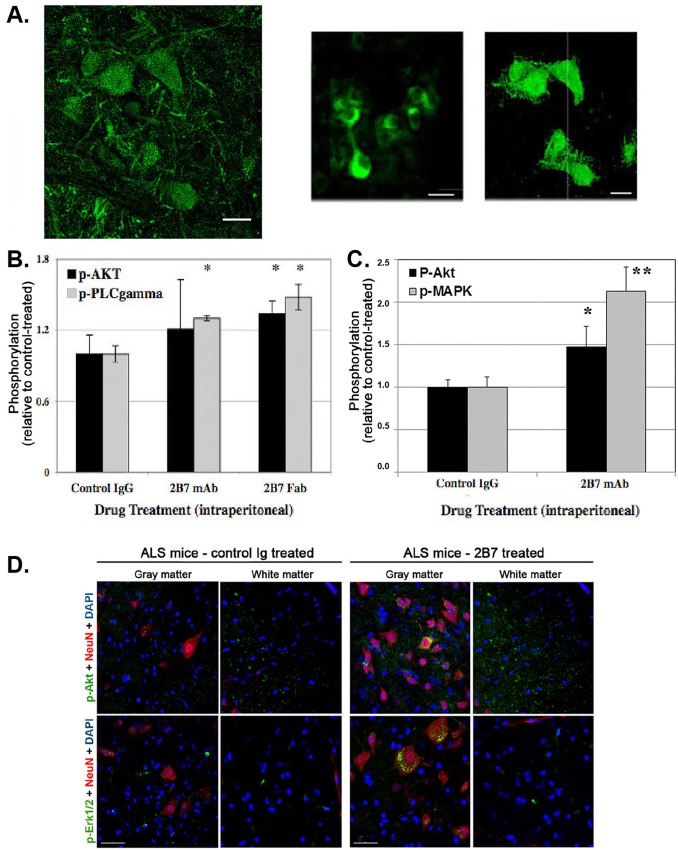
2B7 reaches spinal cord neurons and induces trophic signals in wild type mice and in ALS mice. (**A**) 2B7 was labeled with Atto-488 fluorochrome and 15 μg 2B7-ATTO were injected intraperitoneally into 6-week old C57BL/6J wild type mice. After 48 hrs, mice were perfused, and sections of their spinal cord examined. Staining was observed in ventral motor neurons of lumbar spinal cord (smaller pictures showing only one layer), and in confocal images (large picture on left). The label within subcellular organelles can be detected, indicative of internalized fluorochrome within vesicles. Motor neurons at all levels of the spinal cord contained the label. (**B**) A single intraperitoneal injection of 2B7 was done in wild type C57BL/6J mice (n = 3 per group), spinal cords were collected after 18 hrs, and detergent lysates of soft tissues were prepared and studied by Western blots. Quantification of p-Akt or p-PLCγ was done by densitometric analysis *versus* actin to standardize loading. The data shows activated p-Akt, and p-PLCγ in 2B7–treated mice, when compared to control IgG–treated mice. t-test * p<0.05 versus control. (**C**) The quantitative data in (B) was validated using symptomatic ALS mice (post-natal day 102). ALS mice received a single intraperitoneal injection of 2B7 or non-binding control IgG (n = 5 per group). After ~18 hrs spinal cords were collected for biochemical and immunohistochemical studies. Detergent lysates of soft tissues were studied by Western blots using with anti-p-Akt, anti-p-MAPK, or anti-actin to standardize loading. The data suggests activated p-Akt, and p-MAPK in 2B7–treated ALS mice, when compared to control IgG–treated ALS mice. t-test * p<0.05 and ** p<0.01 versus control. (**D**) Immunohistochemical studies of ALS spinal cords. Cryosections of 2B7-treated or control-treated ALS mice (as in panel C) were immunostained with anti-p-Akt and anti-p-MAPK antibodies. Fluorescent microscopy data shows that 2B7-treatment induced Akt activation in neuronal cell bodies in the gray matter (co-localized with the NeuN marker) and in neuronal fibers of the white matter; and MAPK activation almost exclusively in neuronal cell bodies, but not in neuronal fibers of the white matter. Akt and MAPK activation in neurons are not seen in control IgG-treated ALS samples.

We studied whether 2B7 administered at the periphery can reach the motor neuron cell body in the spinal cord, a key feature for neurotrophic activity [29]. Labeled 2B7 was administered intraperitoneally to wild type mice, and after 48 hours spinal cords were studied for the presence of labeled 2B7. The 2B7 signal was found in the cell bodies of putative ventral motor neurons of lumbar spinal cord (**Fig 6A**), and there was no label in any other cells within the spinal cord.

Then, we asked whether activation of TrkC-FL signals is detected in spinal cord after 2B7 treatment. Wild type mice received single intraperitoneal injection of 2B7 Fab or control (~30 μg/mouse), and ~18 hours later the mice were saline-perfused, spinal cord tissues were collected, and activation of trophic signals were quantified by western blots (n = 3 mice per group). From densitometric quantification, the data shows that 2B7 treatment activated a significant ~35% increase in p-AKT and a significant ~50% increase in p-PLCγ compared to control treatment (**Fig 6B**). Note that these studies were done in freely moving wild type healthy mice, which have normal motor neuron activity. Therefore the significant increases reported are *above* normal signals.

To complement this study in wild type mice, a study evaluated activation of signals in the spinal cord of ALS mice at post-natal day 102 (symptomatic, n = 5 mice per group). Data shows activated p-Akt and p-MAPK in 2B7–treated mice, when compared to control IgG–treated mice (**Fig 6C**) at ~18 hours post-injection. From these 2B7–treated or control–treated ALS spinal cords, cryosections were prepared for immunohistochemical studies with anti-p-Akt and anti-p-MAPK. The representative fluorescent microscopy data shows that 2B7 activates (*i*) p-Akt in neuronal cell bodies in the gray matter (co-localized with the NeuN marker) and in neuronal fibers of the white matter; and (*ii*) p-MAPK almost exclusively in neuronal cell bodies, but not in neuronal fibers of the white matter. In contrast, control-treated ALS mice studied in parallel had lower or absent neuronal Akt and MAPK activation (**Fig 6D**).

Taken together, these data demonstrate that 2B7 treatment activates trophic signals significantly above normal homeostatic levels in healthy wild type mice and in spinal cord neurons of symptomatic ALS mice. This suggests that therapy may be initiated after the onset of symptoms. Moreover, 2B7 treatment benefits from the key features of a relatively simple systemic administration.

### Criteria for therapeutic dose

A dose and frequency for a therapeutic study in ALS mice was estimated from the pharmacokinetic study. A relatively low dose and frequency of drug were chosen from the pharmacokinetic data. The rationale was based on the following reasons. *First*, the schedule has to be acceptable for pharmacological treatment of chronic diseases such as ALS. *Second*, biochemical studies in cultured cells confirmed that TrkC activation by the agonists was long-lived. *Third*, biochemical studies *in vivo* showed that within 18 hours of injection there was activated TrkC in the spinal cord, and imaging studies showed that at 48 hours after injection the reagents were still present in spinal cord neuronal cell bodies. *Fourth*, a receptor agonist should not require saturation of all the receptor sites to activate signals, and a low level of agonist in the circulation could activate TrkC without causing receptor desensitization or down-regulation (which would be counterproductive). *Fifth*, pharmacokinetic experiments showed reasonable stability in circulation after intraperitoneal injection.

### 2B7 Fabs extend life span, improve motor neuron performance, body weight, and motor neuron phenotype in a mouse model of ALS.

A 0.5 mg/kg dose of 2B7 Fab or control non-binding IgG at a frequency of three times a week was injected intraperitoneally in transgenic SOD1 G93A mutant mice.

For the therapeutic study, we elected to use 2B7 Fabs (lacking the Fc domain) to eliminate the potential for immune activation that could lead to misinterpretation. For example, we previously reported [15] that mAb 2B7 IgG could cause cell death *in vivo* but without detectable binding to TrkC.T1. In retrospect neuronal death was likely caused by Fc receptor-binding, activation of the complement cascade, and/or recruitment of macrophages.

**Fig 7:**
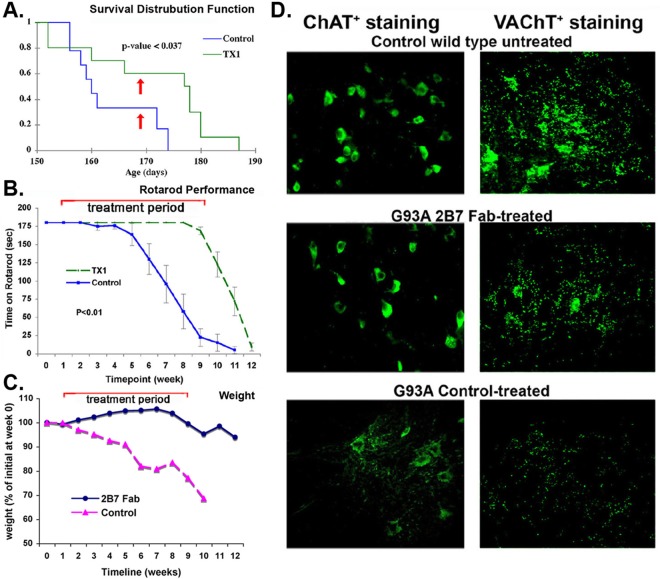
2B7 Fabs extend life-span and improve motor neuron performance and phenotype in a mouse model of ALS. In transgenic SOD1 G93A mutant mice, non-binding control Ig or 2B7 Fabs were injected intraperitoneally 3x/week at 0.5 mg/kg (~10 μg per dose). Treatments were initiated at day ~100 and continued until day ~168. Experiments were done double-blinded, and 2B7 FAb was coded TX1. n = 12 mice per group. The *p* values in survival and Rotarod Kaplan-Meier plots are derived from log-rank tests.(**A**) 2B7 Fab treatment significantly extended life-span, p<0.037 *versus* control. The time at which therapy ended is indicated by red arrows. (**B**) 2B7 Fab treatment significantly improved Rotarod performance. Shown are 1x per week measurements starting at post-natal day ~100, p<0.01 *versus* control. The time-course of drug-treatment is indicated by the red line. (**C**) 2B7 Fab treatment improved or maintained body weight. Shown are 1x per week measurements starting at post-natal day ~100. The time-course of drug-treatment is indicated by the red line. (**D**) The spinal cords of wild type control, and 2B7-treated or Ig-treated ALS mice were dissected at day 160 (n = 2 per group). Cryosections were studied using fluorescent microscopy after immunostaining with anti-ChAT antibodies (a marker of motor neuron cell body) or anti-VAchT antibodies (a marker of motor neuron cell body and fibers). The ChAT and VAchT phenotype appears to be improved in 2B7-treated G93A ALS mice.

In a realistic therapeutic paradigm treatment was initiated at postnatal day ~100 (after disease onset, when hind leg reflexes were compromised). Treatment was continued until postnatal day ~168. At postnatal day 160 the control group had 50% surviving mice whereas the 2B7 Fab group had 80% surviving mice. At postnatal day 168 (when treatment was discontinued) the control group had 35% surviving mice whereas the 2B7 Fab group had 60% surviving mice. At postnatal day 175 the control group had 0% surviving mice whereas the 2B7 Fab group had 60% surviving mice (**Fig 7A**). We note that the surviving mice in the 2B7 Fab group died precipitously between days 177 and 180 (9 days after discontinuation of treatment), and this is coherent with drug elimination pharmacokinetics.

In addition to significantly increased survival (**Fig 7A**), the 2B7 Fab-treated group exhibited significant (p < 0.01) improvement to motor performance in the rotarod test (**Fig 7B**), and maintained body weight (**Fig 7C**). In addition, their spinal cord neurons had enhanced ChAT and VAChT phenotype compared to control-treated ALS mice (**Fig 7D**).

In sum, 2B7 Fab therapy was effective *in vivo* in a mouse model of ALS, even when administered after disease onset, demonstrating that selective TrkC-FL agonists can be significantly and meaningfully effective *in vivo*, improving the key endpoints of life-span, motor function, weight, and motor neuron phenotype. To our knowledge, no reported therapeutic treatment has achieved such efficacy in these endpoints in this animal model.

## Discussion

This work addresses the concept that functional ligands can selectively target the ectodomain of receptors that have identical primary sequences and which are not discriminated by natural ligands. Extension of this work to other receptors known to generate intracellular isoforms would generalize the concept and help to validate important therapeutic targets.

### Structural differences in TrkC-FL and TrkC.T1 ectodomain

To our knowledge, this is the first example of a mAb differentiating between naturally occurring RTK ectodomains with identical primary structure. Moreover, the antibody acts as a functional agonist of TrkC-FL, while the natural ligand NT-3 does not discriminate between isoforms. Our strategy is therefore distinct from other mAb-based ligands that target unique structures generated by activating mutations in a receptor ectodomain [23].

The 2B7 epitope may be defined as a “receptor hot spot”, and it was surprising that it would be expressed as a “cryptic epitope” in the TrkC.T1 isoform. The functional relevance of conformation and “cryptic epitopes” has been documented for Trk-receptors. For example, it is possible to activate Trk with monvalent/monomeric ligands through putative conformational mechanisms [26,30], rather than by directly inducing or stabilizing Trk-Trk dimers [23]. “Agonistic hot spots” in Trk receptors can be unmasked by homotypic co-expression of p75^NTR^ [31], and these hot spots can be regulated functionally by antibody-based ligands [32]. Overall, these previous results support the notion of conformational regulation, through mechanisms that can be ligand-dependent.

The present work also demonstrates a third mechanism: that the gain of a neoepitope in the intracellular domain may be instructive of extracellular domain protein folding. It will be important to identify how the 2B7-recognized structure (TrkC-FL; TrkC.T1 ^Δinsert^) and the 2B7-unrecognized structure (TrkC.T1) are formed, because it may be relevant to many receptors that have splicing isoforms. Current work addresses this issue, under the hypothesis of differential folding and S-S formation during Golgi-ER transport.

We show that ligand-dependent signals differ between TrkC-FL and TrkC.T1, and it is also possible that functional differences may be independent of a conventional ligand. Synaptogenesis during development is regulated by physical interactions of TrkC.T1 with PTPsigma and TrkC-FL appears to be less effective at this role [33]. Potentially the structural differences at the ectodomains that we report here may contribute to specific physical interactions with PTPsigma and we are currently exploring this hypothesis.

A strategy that simultaneously affords intracellular truncation together with conformational changes to the ectodomain may be an efficient use of a single gene to generate proteins with different signal transduction properties (e.g. lacking a tyrosine kinase domain) and which can also differentially interact with non-conventional “ligands” or co-receptors through homotypic interactions (e.g. p75^NTR^ on the same cell membrane) or heterotypic interactions (e.g. PTPsigma on other cells), or to act as a conventional “dominant negative” inhibitor.

### Receptor isoforms and disease

We used TrkC-FL agonist mAb 2B7 to validate selective agonist-dependent activation of this target as a potential therapeutic strategy for ALS. Moreover, we showed the mechanism of action by which TrkC.T1 plays a neurotoxic role in ALS, and suggest that TrkC.T1 antagonists or silencing may be potential therapeutics. Our results raise caution about the therapeutic use of growth factors that can activate receptor isoforms, and specifically show the significant barriers to the use of neurotrophins as drugs.

### p75^NTR^ receptors as therapeutic barriers

We conclude that avoiding engagement of p75^NTR^ and TrkC.T1 seems essential in any therapeutic strategy. Neurotrophins bind to p75^NTR^, which has multiple actions that include pro-death and pro-inflammatory signals [13]. p75^NTR^ has widespread expression in motor neurons [34], sensory and sympathetic neurons [35–37], and non-neuronal subtypes such as Schwann cells [38,39]. The p75^NTR^ is re-expressed during/following injury to recapitulate its developmental role in axonal pruning and cell death [32], and the cleaved extracellular domain of p75^NTR^ is a biomarker in ALS [40,41].

In sum, p75^NTR^ expression in cells not meant to be targeted and up-regulation in disease would compromise the use of wild type neurotrophins. Indeed, p75^NTR^ antagonists have modest efficacy at delaying symptoms in ALS models, and engineered neurotrophins that do not bind to p75^NTR^ are neuroprotective whereas wild type neurotrophins are not [13,14].

### TrkC.T1 receptors as therapeutic barriers

Biochemical and cell biological data indicate that TrkC.T1 signals differ from TrkC-FL [42] and can activate RAC1 in a ligand-dependent manner [43]. Genetic and pharmacological data indicate that truncated TrkC.T1 is deleterious to neurons but the mechanism of action was unclear [15]. Here we extend those studies to show that TrkC.T1 causes a paracrine neurotoxic damage by directly up-regulating TNF-α, most likely through RAC1 activation, which is reported to up-regulate TNF-α.

Given the known role of TNF-α in human ALS [10], our data showing the role of TrkC.T1 at increasing neurotoxic TNF-α, and the correlation of TrkC.T1 up-regulation associated with down-regulation of miR-128 and disease progression in human sporadic ALS and in an ALS animal model, we suggest that TrkC.T1 may be a potential target for ALS therapy. This could be achieved by using TrkC.T1 antagonists, Rac1 inhibitors, by silencing TrkC.T1 expression using our pLKO.1^TrkC-T1^ vectors, or by re-expressing miR-128 to promote degradation of TrkC.T1 mRNA. Current work is addressing these possibilities.

### Poor pharmacokinetics and delivery as therapeutic barriers

Neurotrophins have very short half-lives in circulation [11]. The poor pharmacokinetics and poor specificity of NT-3, combined with the inability of NT-3 to reach the vast expanse of target receptors (from neuromuscular junctions to motor neuron cell bodies in spinal cord behind the blood brain barrier) makes them unsuitable as systemic drugs, and poor therapeutic agents for ALS. Experimental treatments using NT-3 protein or viral vectors expressing NT-3 (as well as therapies using many other growth factors) have generally failed for long-term therapy of ALS and failed primary endpoints in human clinical trials [44,45].

Although NT-3 delivered to spinal cord could spare neurons in spinal cord, it did not improve function or survival [14]. Likewise, adenoviruses expressing high levels of NT-3 injected in muscle protected the innervation of that specific muscle but did not protect centrally [6]. Another growth factor, IGF-1, also failed in ALS when injected subcutaneously [17] or when expressed in muscle [17], but had some efficacy after intraspinal delivery using viral vectors [46].

These results are consistent with the fact that signals at the neuromuscular junction differ from signals at the cell body [47] and emphasize the importance of biodistribution and pharmacokinetics to regulate the “location and tempo” of receptor activation required for neuronal survival [48]. Direct and repeated injection of therapeutics in each muscle or in spinal cord is problematic because of the large expanse that would need to be injected. A suitable therapy would have to reach all target tissues at anatomically distant places, from a single delivery route. MAbs as therapeutics are spared most or all of these problems.

### TrkC-FL as a therapeutic target

MAbs are known to be relatively stable in circulation and mAb 2B7 and Fabs have a relatively long circulating half-life, are bioavailable and can be detected in motor neurons of the PNS and the CNS after a single systemic delivery. Additionally, 2B7 and Fabs are selective agonists for the target TrkC-FL. Hence, we used a challenging therapeutic paradigm with treatment initiated after first signs of motor decline were detected. This is a realistic model because patients would present with existing symptoms.

The therapeutic doses and frequency of 2B7 Fabs utilized are acceptable for treatment of chronic diseases such as ALS. We rationalized a dose and frequency suitable to achieve and maintain low-levels of circulating agonist, which would be sufficient to maintain a constant pro-survival signal without causing desensitization.

While antibodies have been shown to be high-quality therapeutics for cancer and immune disorders, the use of an agonist of an RTK also requires caution because of the potential for oncogenicity. Indeed, TrkC-FL has roles in tumor development, including breast cancer and adenoid cystic carcinoma, which could be a safety concern. On the other hand, targeting TrkC-FL therapeutically could potentially extend to neurodegenerative pathologies other than ALS where TrkC-FL and NT-3 are relevant, such as proprioceptive neurons, the repair of inner ear neurons damaged by noise, amongst others. Hence, it will be crucial to carry out detailed pharmacokinetic and pharmacodynamic studies to optimize intervention for chronic neurodegeneration.

The results presented here may guide the development of pro-neuroprotective (TrkC-FL) and anti-neurotoxic (TrkC.T1) therapeutic strategies in ALS and related diseases.

## Materials and Methods

### Cell lines

Established cell lines HEK293 or NIH-3T3 were transfected with plasmids encoding human or rat full-length *TrkC* (293-TrkC-FL, NIH-TrkC-FL) or coding for human or rat *TrkC*.*T1* (293-TrkC.T1, NIH-TrkC.T1), or coding for TrkC.T1 with the neo-epitope deleted (293-TrkC.T1 ^Δinsert^). Stably transfected cell lines that express high levels of TrkC-FL or TrkC.T1 receptors were generated and subcloned under drug selection (depending on the vector, 0.5 mg/ml G418, or 2 mg/ml puromycin, or 10 mg/ml blastocidin). The HEK293 cell line expressing the human TrkC.T1 ^Δinsert^ construct was generated by transfection with a pcDNA6 blasticidin-resistant plasmid containing a PCR-generated amplicon encompassing amino acids 1 to 528 of human TrkC.T1. The rat glial cell line, rMC-1, has been previously characterized [49].

### Antibodies and Proteins

The mAb 2B7 is an agonistic anti-TrkC IgG-C2 (D5) domain antibody. Production and purification of mAB 2B7 and its Fabs were performed as previously reported [7,50]. The epitope is conserved in mouse, rat and human TrkC and the mAb is known to bind to these species. mAb 2B7 does not bind to p75^NTR^, TrkA, TrkB, and many other cell surface receptors tested. MAb 2B7 binds to cells transfected to express TrkC, but not to untransfected parental cells [6].

The mouse polyclonal 750 antibody was developed in our laboratory against a peptide comprising a neo-epitope which is present only in TrkC.T1 due to mRNA splicing (residues 595–611 sequence GIYVEDVNVYFSKGRHG). The anti-GFAP is a rabbit polyclonal from Millipore. NGF, proNGF and NT-3 were purchased (Alomone Labs and ProSpec-Tany). The rabbit anti-VAChT antibody was a gift from Dr. Claudio Cuello (McGill University) and the rabbit anti-ChAT antibody was purchased (ThermoFisher).

### RNAi knockdown of TrkC.T1

A short hairpin RNA (shRNA) specifically targeting a unique 3’ sequence of the TrkC.T1 mRNA was designed using the DSIR algorithm (http://biodev.cea.fr/DSIR/DSIR.html). The TrkC.T1-targeting shRNA sequence (GGACAATAGAGATCATCTAGT), or a scrambled control sequence (CCTAAGGTTAAGTCGCCCTCG), were cloned into a pLKO.1 lentiviral shRNA-expression vector. pLKO.1^scrambled^ and pLKO.1^TrkC-T1^ lentiviral vectors were packaged in HEK-293T cells and active viral particles were purified. rMC-1 cells were then transduced with lentiviral particles, and selected with 1 μg/ml puromycin. TrkC.T1-specific depletion was determined by real-time quantitative PCR and by Western blotting.

### Expression of TNF-α, and TrkC isoforms

#### ALS mice

For mRNA expression, total RNA, including short RNAs, were extracted from whole spinal cords (WT mice or ALS pre-symptomatic or symptomatic mice) using the miRNeasy® Mini Kit (QIAGEN). One μg of extracted RNA was reverse transcribed with the SuperScript III First-Strand Synthesis System for RT-PCR (Invitrogen). mRNA levels of TrkC isoforms and TNF-α were analyzed by real-time quantitative PCR using SYBR Green I (Quanta) in an 7500 FAST real-time PCR thermocycler (Applied Biosystems) and thermal cycling was performed as follows: 94° C for 30 sec, followed by 40 cycles at 94° C for 5 sec, 60° C for 30 sec. Specific primers were designed for the two TrkC isoforms and TNF-α. GAPDH or β-tubulin III were used as housekeeping genes (**Table 1**). Data are expressed as the mean relative quantification (RQ) ± SEM (n = 8 individual mice per group). WT samples were used as a calibrator (RQ = 1).

**Table 1:** Sequences of the primers.

Genes	Amplicants bp
**Mouse TrkC-FL**	**173**
Forward: 5'- -3' TGATCCTCGTGGATGGACAG	
Reverse: 5'- -3'CTTCACTAGTAGATTGGCTCC	
**Mouse TrkC.T1**	**517**
Forward 5'- -3' CCACTTCCTGAAGGAGCCCT	
Reverse 5'- -3' CCCACTCTGGACCTCAGGTT	
**Human TrkC.T1** ^Δ**insert**^	**527**
Forward: 5'- -3'CCCCTCGAGCCACCATGGATGTCTCTCTTTGCCCA	
Reverse: 5'- -3'CTTACCGGTCTACGTGTCCGGCTTGTGGCAGT	
**Mouse TNF-α**	**235**
Forward 5'- -3'GAGTCCGGGCAGGTCTACTTT	
Reverse 5'- -3'CAGGTCACTGTCCCAGCATCT	
**Mouse Beta-Tubulin III**	**160**
Forward 5'- -3' CTCAGGGGCCTTTGGACATC	
Reverse 5'- -3' CAGGCAGTCGCAGTTTTCAC	
**Mouse GAPDH**	**452**
Forward 5'- -3' ACCACAGTCCATGCCATCAC	
Reverse 5'- -3' TCCACCACCCTGTTGCTGTA	
**Rat TrkC.T1**	**101**
Forward 5'- -3' GTCCAGAGTGGGGATGTGTC	
Reverse 5'- -3' CCATGGTTAAGAGGCTTGGA	
**Rat TNF-α**	**159**
Forward 5'- -3' CTATGTGCTCCTCACCCACA	
Reverse 5'- -3' TGGAAGACTCCTCCCAGGTA	
**Rat GAPDH**	**181**
Forward 5'- -3' AGCCCAGAACATCATCCCTG	
Reverse 5'- -3' CACCACCTTCTTGATGTCATC	

#### HEK293 cells

For quantification of TrkC protein, detergent lysates of 293-TrkC or 293-TrkC.T1 or TrkC.T1 ^Δinsert^ cells were analyzed by Western blotting with MAb 2B7, or antibody 750 specific for TrkC.T1 or Ab C44H5 for all isoforms. For analyses of cell surface expression, 293-TrkC or 293-TrkC.T1 cells were fixed and immunostained with primary mAb 2B7, or antibody 750, or with non-binding mouse IgG control. DAPI was used to stain nuclei. Immunofluorescent pictures were taken using a Leica confocal microscope with 63x magnification.

#### RMC-1 cells

Non infected rMC-1 or cells infected with lentiviral pLKO.1^scrambled^ or pLKO.1^TrkC.T1^ were treated with 2B7 100 nM or NT-3 10 nM or LPS 1 μg/ml for 6 h. Quantification of TNF-α mRNA was performed by real-time quantitative PCR as described previously and primers used for rat TNF-α and rat GAPDH are reported (**Table 1**). Quantification of TNF-α in supernatant (conditioned media) was done using commercial rat TNF-α Standard ELISA (PeproTech). Data are expressed as the mean + SEM relative to the untreated (3 independent experiments, each in triplicate). Cell-associated levels of TNF-α were studied in the same cultures of rMC-1 cells treated as above, by immunofluorescence. Cells were fixed and immunostained with primary rabbit anti-mouse TNF-α (Millipore) followed by incubation with secondary anti-rabbit Alexa^488^ (Invitrogen). Immunofluorescent pictures were taken using a Leica confocal microscope with 63x magnification to reveal cell-associated TNF-α protein (surface, intracellular, and vesicular).

### MicroRNA expression

MicroRNA cDNA synthesis was performed with the qScript microRNA cDNA synthesis kit (Quanta) using 1 μg of total spinal cord RNA. Then, a real time SYBR Green qRT-pCR amplification of miRNAs were performed using 200 nM of each Perfecta microRNA assay primers for miR-128-1 (destabilizes TrkC.T1), miR-151-3p (destabilizes TrkC-FL) [51], or RNU6 (as reference) and universal primers (Quanta). qRT-PCR was performed using an 7500 FAST real-time PCR thermocycler (Applied Biosystems) and thermal cycling was performed as follows: pre-incubation/activation; 95° C for 2 min, followed by 40 cycles at 94° C for 5s, 60° C for 30s. The WT mRNA sample was used as a calibrator (RQ = 1). Data are expressed as the mean + SEM from 6 individual mouse spinal cords of ALS or WT.

### Human spinal cord samples

Samples were collected post-mortem under a protocol from the Institutional Review Ethic Board (Brain Research Centre, UBC), with written informed consent from the donor or the next of kin. The Institutional Review Board specifically approved this study. Samples from four individual spinal cords of non-SOD1-related sporadic ALS (SALS) and three individual non-ALS controls were used. Information on human donors is listed in **Table 2**. Total RNAs were purified form the cervical region, and studies were done by RT-PCR as above. Data are expressed as the mean + SEM with control mRNA samples as a calibrator (RQ = 1).

**Table 2:** ALS patient and Normal Human post mortem spinal cord samples.

Sample ID	Disease status	Region	Sex	Age
VA06-60	nonSOD1 SALS	Cervical	M	66
VA06-11	nonSOD1 SALS	Cervical	F	59
VA02-58	nonSOD1 SALS	Thoracic	M	73
NR09-102	nonSOD1 SALS	Thoracic	F	39
VF07-2594	Normal	Cervical	M	55
VA10-17	Normal	Cervical	F	76
VF07-2593	Normal	Cervical	M	38

### Fluorescent Activated Cell Scan (FACS) analysis

FACs analyses were performed as described [52]. Briefly, cells were resuspended in 0.1 mL of binding buffer were incubated with mAb 2B7 or control mIgG for 20 min at 4°C, washed in binding buffer to remove excess primary antibody, and immunostained with FITC-mIgG secondary antibody for 20 min at 4°C. Cells were acquired and analyzed on a FACScan-BD Sciences using the Cell Quest program. As negative controls, no primary (background fluorescence) or irrelevant mouse IgG (Sigma) were used followed by secondary antibody. Specificity was gauged using various cells expressing different receptors.

### Mild reduction of samples, and biochemical analyses

2% NP-40 detergent lysates were prepared from live 293-TrkC-FL or 293-TrkC.T1 cells. Cleared whole cell lysates were resuspended in SDS-PAGE Laemmli buffer lacking reducing agents. After 1 min of exposure to 90°C, samples were aliquoted equally and the indicated final molar concentration of 2-Mercaptoethanol was added to each aliquot. After 15 min at room temperature, replicate samples were exposed again to 90°C for 1 min, others were not. All samples (0.75x10^6^ cell equivalents/lane) were resolved immediately by SDS-PAGE. After western transfer, membranes were immunoblotted with mAb 2B7 (TrkC-FL specific) or with mAb C44H5 (total TrkC). Equal loading in all lanes was verified, n = 3 independent experiments.

### Cell Metabolism/Survival Assays

The growth/survival profile of the cells were quantified in 96-well plates using the tetrazolium salt reagent 4-(4,5-dimethylthiazol-2-yl)-2,5-diphenyltetrazolium bromide (MTT; Sigma) 48 hr after plating; by reading the optical density (OD), as previously described [46]. Cells cultured in serum-free-medium (SFM) die by apoptosis, but they can be rescued if they express TrkC-FL and are supplemented with NT-3. Cells received vehicle, or were supplemented with 2B7 or 2B7 fabs (tests) or mIgG (negative control) or NT-3 (positive control) at the indicated concentrations. Cellular controls used cells expressing a related receptor TrkA, and its ligand NGF was the positive control. TrkA-expressing cells do not respond to 2B7 (data not shown). All assays were in quadruplicate and were repeated n>3 independent times. MTT data are standardized to optimal dose of neurotrophin = 100% survival, and serum-free medium (SFM) = 0% survival, using the formula ((ODtest − ODSFM) x 100 / (ODoptimal NTF − ODSFM)).

### Kinetics of activation of pro-survival signaling by 2B7 and 2B7 fabs *in vitro*

293-TrkC-FL cells were cultured with 2B7 mAb (50 nM), 2B7 Fab (100 nM), NT-3 (4 nM) or control mIgG (100 nM) for 10 min. Detergent lysates were analyzed by Western blotting with anti-pTyr mAb 4G10 or anti-phospho-MAPK or anti-phospho-Akt (Cell Signaling). After stripping, membrane was re-probed with anti-actin (Sigma) to standardize loading. Quantification was done after densitometric analysis, averaging n = 3 independent experiments standardized to controls (untreated or optimal NT-3 treatment).

### Activation of TrkC-FL in spinal cord *in vivo*

A single intraperitoneal injection of 0.5 mg/kg 2B7 (~10 μg) was done in wild type C57-BL6 mice (n = 3 per group) or ALS mice (n = 5 per group), and tissue was collected ~18 hours post-injection of 2B7 or control IgG. Detergent lysates were analyzed by Western blotting with Anti-phospho-MAPK, anti-phospho-Akt, or anti-p-PLCγ (all from Cell Signaling). After stripping, membranes were re-probed with anti-actin (Sigma) to standardize loading. Quantification was done by densitometric analysis.

### Pharmacokinetics and detection of 2B7 in the spinal cord

The pharmacokinetics of 2B7 or 2B7 Fabs were studied after intraperitoneal administration in a 6-week old C57BL/6J (B6). The circulating levels of 2B7 or 2B7 Fabs able to bind TrkC were quantified by ELISA based on specific binding to the known peptide epitope. Blood was collected at different times after a single IV injection (100 μg), plasma was prepared in Heparin-coated tubes, and assayed in ELISA. Pre-bleed was used as background control.

For studies of 2B7 reaching motor neuron cell bodies in spinal cord, labeled mAb (2B7-ATTO) was injected intraperitoneally into a 6-week old C57BL/6J (B6) mice in two doses of 75 μg, 12 hr apart. After 48 hr, mice were perfused, and sections of their spinal cord were examined by confocal microscopy.

### 2B7 Fabs therapeutic efficacy in SOD1 mice

B6.Cg-Tg(SOD1*G93A)1Gur/J (JAX Labs, stock # 004435) mice over expressing the human SOD1 gene with the G93A mutation, crossed into a CBL/black 6 background, an average 50% survival at 157.1 ± 9.3 days [1]. The agonist was injected intraperitoneally three times a week at 0.5 mg/kg 2B7 Fab (~10 μg/mouse/injection), in transgenic SOD1G93A mutant mice (n = 12 mice per group, 6 males and 6 females). Treatment was initiated after disease onset was measured (e.g. when hind leg reflexes were compromised, at postnatal day ~100). Treatment was continued until postnatal day 168. These experiments and the quantification of experimental endpoint were done double-blinded.

### Experimental endpoints in ALS

Hindlimb leg reflex (HLR) is measured as a reduction in hindlimb extension when animals are lifted by the tail, and is an early deficit observed in mutant SOD1 Tg mice. Animals are lifted by the base of the tail to score hindlimb extension and postural reflexes [9]. Experimental endpoints of body weight, performance in the rotarod (measurements weekly starting at day ~100, when hind-leg reflexes were compromised), and overall survival (extension of life-span) were done as described [53] for mutant SOD1 Tg mice ± mAb 2B7 ± control treatment. The health of the animals was monitored daily, and there were no unexpected events.

### Study Approval

All animal protocols and endpoints in these studies were specifically approved by the University of British Columbia and McGill University IACUC. Humane endpoints were used during the animal survival study as described [23], and mice were euthanized when they met the specified criteria of high loss of body weight, immobility, or Hind-leg reflex level 4 as defined in the IACUC approved protocols. Euthanasia was done by CO_2_ and cervical dislocation.

## References

[1] Brock JH, Rosenzweig ES, Blesch A, Moseanko R, Havton LA, et al (2010) Local and remote growth factor effects after primate spinal cord injury. J Neurosci 30: 9728–9737. 10.1523/JNEUROSCI.1924-10.2010 20660255 PMC2927098

[2] Sendtner M, Holtmann B, Hughes RA (1996) The response of motoneurons to neurotrophins. Neurochem Res 21: 831–841. 10.1007/bf02532307 8873088

[3] Saragovi HU, Hamel E, Di Polo A (2009) A neurotrophic rationale for the therapy of neurodegenerative disorders. Curr Alzheimer Res 6: 419–423. 10.2174/156720509789207912 19874265

[4] Mitchell JD, Borasio GD (2007) Amyotrophic lateral sclerosis. Lancet 369: 2031–2041. 10.1016/S0140-6736(07)60944-1 17574095

[5] Hurko O, Walsh FS (2000) Novel drug development for amyotrophic lateral sclerosis. J Neurol Sci 180: 21–28. 10.1016/s0022-510x(00)00419-6 11090860

[6] Haase G, Pettmann B, Vigne E, Castelnau-Ptakhine L, Schmalbruch H, et al (1998) Adenovirus-mediated transfer of the neurotrophin-3 gene into skeletal muscle of pmn mice: therapeutic effects and mechanisms of action. J Neurol Sci 160 Suppl 1: S97–105. 10.1016/s0022-510x(98)00207-x 9851658

[7] Park S, Kim HT, Yun S, Kim IS, Lee J, et al (2009) Growth factor-expressing human neural progenitor cell grafts protect motor neurons but do not ameliorate motor performance and survival in ALS mice. Exp Mol Med 41: 487–500. 10.3858/emm.2009.41.7.054 19322031 PMC2721146

[8] Zhou L, Baumgartner BJ, Hill-Felberg SJ, McGowen LR, Shine HD (2003) Neurotrophin-3 expressed in situ induces axonal plasticity in the adult injured spinal cord. J Neurosci 23: 1424–1431. 12598631 10.1523/JNEUROSCI.23-04-01424.2003 PMC6742279

[9] Segal RA (2003) Selectivity in neurotrophin signaling: theme and variations. Annu Rev Neurosci 26: 299–330. 10.1146/annurev.neuro.26.041002.131421 12598680

[10] Ibanez CF, Simi A (2012) p75 neurotrophin receptor signaling in nervous system injury and degeneration: paradox and opportunity. Trends Neurosci 35: 431–440. 10.1016/j.tins.2012.03.007 22503537

[11] Shepheard SR, Chataway T, Schultz DW, Rush RA, Rogers ML (2014) The extracellular domain of neurotrophin receptor p75 as a candidate biomarker for amyotrophic lateral sclerosis. PLoS One 9: e87398 10.1371/journal.pone.0087398 24475283 PMC3903651

[12] Josephy-Hernandez S, Jmaeff S, Pirvulescu I, Aboulkassim T, Saragovi HU (2016) Neurotrophin receptor agonists and antagonists as therapeutic agents: An evolving paradigm. Neurobiol Dis. 10.1016/j.nbd.2016.08.004 27546056

[13] Bai Y, Dergham P, Nedev H, Xu J, Galan A, et al (2010) Chronic and acute models of retinal neurodegeneration TrkA activity are neuroprotective whereas p75NTR activity is neurotoxic through a paracrine mechanism. J Biol Chem 285: 39392–39400. 10.1074/jbc.M110.147801 20943663 PMC2998128

[14] Enomoto M, Bunge MB, Tsoulfas P (2013) A multifunctional neurotrophin with reduced affinity to p75NTR enhances transplanted Schwann cell survival and axon growth after spinal cord injury. Exp Neurol 248: 170–182. 10.1016/j.expneurol.2013.06.013 23792206

[15] Bai Y, Shi Z, Zhuo Y, Liu J, Malakhov A, et al (2010) In glaucoma the upregulated truncated TrkC.T1 receptor isoform in glia causes increased TNF-alpha production, leading to retinal ganglion cell death. Invest Ophthalmol Vis Sci 51: 6639–6651. 10.1167/iovs.10-5431 20574020 PMC3055774

[16] Aboulkassim T, Tong XK, Tse YC, Wong TP, Woo SB, et al (2011) Ligand-dependent TrkA activity in brain differentially affects spatial learning and long-term memory. Mol Pharmacol 80: 498–508. 10.1124/mol.111.071332 21616921

[17] Esteban PF, Yoon HY, Becker J, Dorsey SG, Caprari P, et al (2006) A kinase-deficient TrkC receptor isoform activates Arf6-Rac1 signaling through the scaffold protein tamalin. J Cell Biol 173: 291–299. 10.1083/jcb.200512013 16636148 PMC2063819

[18] Dorsey SG, Renn CL, Carim-Todd L, Barrick CA, Bambrick L, et al (2006) In vivo restoration of physiological levels of truncated TrkB.T1 receptor rescues neuronal cell death in a trisomic mouse model. Neuron 51: 21–28. 10.1016/j.neuron.2006.06.009 16815329

[19] Yanpallewar SU, Barrick CA, Buckley H, Becker J, Tessarollo L (2013) Deletion of the BDNF truncated receptor TrkB.T1 delays disease onset in a mouse model of amyotrophic lateral sclerosis. PLoS One 7: e39946 10.1371/journal.pone.0039946 22761934 PMC3384607

[20] LeSauteur L, Maliartchouk S, Le Jeune H, Quirion R, Saragovi HU (1996) Potent human p140-TrkA agonists derived from an anti-receptor monoclonal antibody. J Neurosci 16: 1308–1316. 8778282 10.1523/JNEUROSCI.16-04-01308.1996 PMC6578545

[21] Saragovi HU, Gehring K (2000) Development of pharmacological agents for targeting neurotrophins and their receptors. Trends Pharmacol Sci 21: 93–98. 10.1016/s0165-6147(99)01444-3 10689362

[22] Saragovi HU, Zaccaro MC (2002) Small molecule peptidomimetic ligands of neurotrophin receptors, identifying binding sites, activation sites and regulatory sites. Curr Pharm Des 8: 2201–2216. 10.2174/1381612023393215 12369863

[23] Guillemard V, Ivanisevic L, Garcia AG, Scholten V, Lazo OM, et al (2010) An agonistic mAb directed to the TrkC receptor juxtamembrane region defines a trophic hot spot and interactions with p75 coreceptors. Dev Neurobiol 70: 150–164. 10.1002/dneu.20776 19953569

[24] Watson F, Porcionatto M, Bhattacharyya A, Stiles C, Segal R (1999) TrkA glycosylation regulates receptor localization and activity. J Neurobiol 39: 323–336. 10.1002/(sici)1097-4695(199905)39:2<323::aid-neu15>3.0.co;2-4 10235685

[25] Lee FS, Chao MV (2001) Activation of Trk neurotrophin receptors in the absence of neurotrophins. Proc Natl Acad Sci U S A 98: 3555–3560. 10.1073/pnas.061020198 11248116 PMC30691

[26] Ye H, Kuruvilla R, Zweifel LS, Ginty DD (2003) Evidence in support of signaling endosome-based retrograde survival of sympathetic neurons. Neuron 39: 57–68. 10.1016/s0896-6273(03)00266-6 12848932

[27] Lebrun-Julien F, Duplan L, Pernet V, Osswald I, Sapieha P, et al (2009) Excitotoxic death of retinal neurons in vivo occurs via a non-cell-autonomous mechanism. J Neurosci 29: 5536–5545. 10.1523/JNEUROSCI.0831-09.2009 19403821 PMC6665839

[28] Barcelona PF, Saragovi HU (2015) A Pro-Nerve Growth Factor (proNGF) and NGF Binding Protein, alpha2-Macroglobulin, Differentially Regulates p75 and TrkA Receptors and Is Relevant to Neurodegeneration Ex Vivo and In Vivo. Mol Cell Biol 35: 3396–3408. 10.1128/MCB.00544-15 26217017 PMC4561734

[29] Guidi M, Muinos-Gimeno M, Kagerbauer B, Marti E, Estivill X, et al (2010) Overexpression of miR-128 specifically inhibits the truncated isoform of NTRK3 and upregulates BCL2 in SH-SY5Y neuroblastoma cells. BMC Mol Biol 11: 95 10.1186/1471-2199-11-95 21143953 PMC3019150

[30] Lee F, Chao M (2001) Activation of Trk neurotrophin receptors in the absence of neurotrophins. Proc Natl Acad Sci (USA) 98: 3555–3560. 10.1073/pnas.061020198 11248116 PMC30691

[31] Reichardt LF, Mobley WC (2004) Going the distance, or not, with neurotrophin signals. Cell 118: 141–143. 10.1016/j.cell.2004.07.008 15260984

[32] Maliartchouk S, Saragovi HU (1997) Optimal nerve growth factor trophic signals mediated by synergy of TrkA and p75 receptor-specific ligands. J Neurosci 17: 6031–6037. 9236214 10.1523/JNEUROSCI.17-16-06031.1997 PMC6568372

[33] Lemmon MA, Schlessinger J (2010) Cell signaling by receptor tyrosine kinases. Cell 141: 1117–1134. 10.1016/j.cell.2010.06.011 20602996 PMC2914105

[34] Maliartchouk S, Debeir T, Beglova N, Cuello AC, Gehring K, et al (2000) Genuine monovalent ligands of TrkA nerve growth factor receptors reveal a novel pharmacological mechanism of action. J Biol Chem 275: 9946–9956. 10.1074/jbc.275.14.9946 10744669

[35] Brahimi F, Liu J, Malakhov A, Chowdhury S, Purisima EO, et al (2010) A monovalent agonist of TrkA tyrosine kinase receptors can be converted into a bivalent antagonist. Biochim Biophys Acta 1800: 1018–1026. 10.1016/j.bbagen.2010.06.007 20600627 PMC2943489

[36] Brahimi F, Malakhov A, Lee HB, Pattarawarapan M, Ivanisevic L, et al (2009) A peptidomimetic of NT-3 acts as a TrkC antagonist. Peptides 30: 1833–1839. 10.1016/j.peptides.2009.07.015 19647025 PMC2755609

[37] Chen D, Brahimi F, Angell Y, Li YC, Moscowicz J, et al (2009) Bivalent peptidomimetic ligands of TrkC are biased agonists and selectively induce neuritogenesis or potentiate neurotrophin-3 trophic signals. ACS Chem Biol 4: 769–781. 10.1021/cb9001415 19735123 PMC2756187

[38] Zaccaro MC, Ivanisevic L, Perez P, Meakin SO, Saragovi HU (2001) p75 Co-receptors regulate ligand-dependent and ligand-independent Trk receptor activation, in part by altering Trk docking subdomains. J Biol Chem 276: 31023–31029. 10.1074/jbc.M104630200 11425862

[39] Ivanisevic L, Banerjee K, Saragovi HU (2003) Differential cross-regulation of TrkA and TrkC tyrosine kinase receptors with p75. Oncogene 22: 5677–5685. 10.1038/sj.onc.1206864 12944916

[40] Takahashi H, Arstikaitis P, Prasad T, Bartlett TE, Wang YT, et al (2011) Postsynaptic TrkC and presynaptic PTPsigma function as a bidirectional excitatory synaptic organizing complex. Neuron 69: 287–303. 10.1016/j.neuron.2010.12.024 21262467 PMC3056349

[41] Coles CH, Mitakidis N, Zhang P, Elegheert J, Lu W, et al (2014) Structural basis for extracellular cis and trans RPTPsigma signal competition in synaptogenesis. Nat Commun 5: 5209 10.1038/ncomms6209 25385546 PMC4239663

[42] Yan Q, Johnson EM Jr. (1988) An immunohistochemical study of the nerve growth factor receptor in developing rats. J Neurosci 8: 3481–3498. 2845023 10.1523/JNEUROSCI.08-09-03481.1988 PMC6569432

[43] Kerkhoff H, Jennekens FG, Troost D, Veldman H (1991) Nerve growth factor receptor immunostaining in the spinal cord and peripheral nerves in amyotrophic lateral sclerosis. Acta Neuropathol 81: 649–656. 10.1007/bf00296375 1715633

[44] Pehar M, Cassina P, Vargas MR, Xie Y, Beckman JS, et al (2006) Modulation of p75-dependent motor neuron death by a small non-peptidyl mimetic of the neurotrophin loop 1 domain. Eur J Neurosci 24: 1575–1580. 10.1111/j.1460-9568.2006.05040.x 17004921

[45] Turner BJ, Murray SS, Piccenna LG, Lopes EC, Kilpatrick TJ, et al (2004) Effect of p75 neurotrophin receptor antagonist on disease progression in transgenic amyotrophic lateral sclerosis mice. J Neurosci Res 78: 193–199. 10.1002/jnr.20256 15378612

[46] Franz CK, Federici T, Yang J, Backus C, Oh SS, et al (2009) Intraspinal cord delivery of IGF-I mediated by adeno-associated virus 2 is neuroprotective in a rat model of familial ALS. Neurobiol Dis 33: 473–481. 10.1016/j.nbd.2008.12.003 19135533

[47] Haase G, Pettmann B, Raoul C, Henderson CE (2008) Signaling by death receptors in the nervous system. Curr Opin Neurobiol 18: 284–291. 10.1016/j.conb.2008.07.013 18725296 PMC2668142

[48] Barinaga M (1994) Neurotrophic factors enter the clinic. Science 264: 772–774. 10.1126/science.8171331 8171331

[49] Bartus RT (2012) Translating the therapeutic potential of neurotrophic factors to clinical 'proof of concept': a personal saga achieving a career-long quest. Neurobiol Dis 48: 153–178. 10.1016/j.nbd.2012.04.004 22525569

[50] Wyatt TJ, Rossi SL, Siegenthaler MM, Frame J, Robles R, et al (2011) Human motor neuron progenitor transplantation leads to endogenous neuronal sparing in 3 models of motor neuron loss. Stem Cells Int 2011: 207230 10.4061/2011/207230 21716648 PMC3116523

[51] Sorenson EJ, Windbank AJ, Mandrekar JN, Bamlet WR, Appel SH, et al (2008) Subcutaneous IGF-1 is not beneficial in 2-year ALS trial. Neurology 71: 1770–1775. 10.1212/01.wnl.0000335970.78664.36 19029516 PMC2617770

[52] Messi ML, Clark HM, Prevette DM, Oppenheim RW, Delbono O (2007) The lack of effect of specific overexpression of IGF-1 in the central nervous system or skeletal muscle on pathophysiology in the G93A SOD-1 mouse model of ALS. Exp Neurol 207: 52–63. 10.1016/j.expneurol.2007.05.016 17597610 PMC2043146

[53] Sarthy VP, Brodjian SJ, Dutt K, Kennedy BN, French RP, et al (1998) Establishment and characterization of a retinal Muller cell line. Invest Ophthalmol Vis Sci 39: 212–216. 9430566

